# Nonlinear effects of humidex on risk of outpatient visit for allergic conjunctivitis among children and adolescents in Shanghai, China: A time series analysis

**DOI:** 10.7189/jogh.13.04132

**Published:** 2023-11-03

**Authors:** Han Zhao, Yun Yang, Changming Feng, Wushuang Wang, Chenhao Yang, Yue Yin, Lan Gong, Tong Lin

**Affiliations:** 1 Department of Ophthalmology, Second Xiangya Hospital of Central South University, Changsha, Hunan, China; 2 Hunan Clinical Research Center of Ophthalmic Disease, Changsha, Hunan, China; 3 Department of Ophthalmology, Eye, Ear, Nose, and Throat Hospital of Fudan University, Shanghai, China; 4 Laboratory of Myopia, NHC Key Laboratory of Myopia (Fudan University), Chinese Academy of Medical Sciences, Shanghai, China; 5 Department of Ophthalmology, Children's Hospital of Fudan University, Shanghai, China

## Abstract

**Background:**

Various epidemiological studies have focused on the adverse health outcomes of meteorological factors. However, there has been little research on the impact of humidex on allergic conjunctivitis, especially in child and adolescent populations. We aimed to explore the impact of humidex, a comprehensive index of relative humidity and temperature, on child and adolescent allergic conjunctivitis admissions.

**Methods:**

Outpatient visit data for allergic conjunctivitis, meteorological factors and air pollutants in Shanghai for the 2017-2022 period were retrieved. For the purpose of analysing the nonlinear connection and lag impact between humidex and admissions for paediatric and adolescent allergic conjunctivitis, the distributed lag nonlinear model (DLNM) was fitted.

**Results:**

A total of 147 090 cases were included in our cohort. We found a significantly nonlinear effect on humidex and allergic conjunctivitis. In the single-day lag pattern, the relative risks (RR) of allergic conjunctivitis were significant at lag 0 (RR = 1.08, 95% confidence interval (CI) = 1.05-1.11) to lag 2 (RR = 1.01, 95% CI = 1.00-1.01), lag 5 (RR = 1.01, 95% CI = 1.00-1.01) to lag 9 (RR = 1.01, 95% CI = 1.00-1.01), and lag 14 (RR = 1.02, 95% CI: 1.01-1.03). In the cumulative-lag day pattern, the RR of allergic conjunctivitis were significant at lag 0-0 (RR = 1.08, 95% CI = 1.05-1.11) to lag 0-14 (RR = 1.21, 95% CI = 1.13-1.28). We found that boys, children aged 7-17 years, and children in the warm season were more vulnerable to humidex. In addition, the highest attributable fraction (AF) and attributable number (AN) of humidex are at lag 0-14 (AF = 0.17, AN = 25 026).

**Conclusions:**

Humidex exposure markedly increased the risk of allergic conjunctivitis, especially in highly high humidex. Appropriate public health management is needed for disease management and early intervention.

Allergic conjunctivitis, one of the most prevalent diseases of the conjunctiva, is marked by inflammation of the conjunctiva and intense itching, red eyes, mucus discharge and swollen eyelids. It can disrupt the ocular surface and lead to unsatisfactory quality of life [1]. Allergic conjunctivitis is not a single disease but a collective term for several diseases with differences in immunologic mechanisms, clinical manifestations, therapeutic management and prognosis. It is therefore classified in various ways, including severity, duration and immunologic mechanisms, such as seasonal allergic conjunctivitis (SAC), perennial allergic conjunctivitis (PAC), and atopic keratoconjunctivitis (AKC) [2]. According to published research, 15-40% of Americans might suffer from allergic conjunctivitis [3]. In Shanghai, the total prevalence of allergic conjunctivitis in children was 28% based on the International Study of Asthma and Allergies in Childhood (ISAAC) questionnaire [4]. The World Allergy Organization (WAO) reports that allergic disorders are becoming more common worldwide annually, posing a severe disease burden, especially in paediatric and adolescent populations and developing countries [5]. It most frequently influences children and adolescents, affecting their visual development and increasing family burden [6,7].

The ocular surface is an ideal site for hypersensitivity reactions due to direct exposure to the external environment and the presence of many immune cells and lymphoid tissue locally [8,9]. Existing evidence has indicated that the environment is an essential trigger for allergy disorders and is closely related to it, in addition to the allergic constitution and genetic causes [10,11], suggesting that the primary prevention approach is to keep children and adolescents from environmental risk factors for allergic conjunctivitis.

Meteorological factors are crucial environmental factors associated with human wellness [12,13]. Several studies have demonstrated that meteorological factors are related to allergic diseases, including allergic rhinitis [14], asthma [15,16] and atopic dermatitis [15]. For instance, Khalaila et al. observed that visits to the emergency department for conjunctivitis were related to the ambient temperature [17]. In addition, a nationwide analysis showed that mean temperature, precipitation, and relative humidity were correlated with outpatient visits to acute haemorrhagic conjunctivitis [18]. Recently, several studies have suggested that air temperature as well as wind speed positively correlate with allergic conjunctivitis [19,20]. Several previous studies have evaluated the relationship between humidity and allergic conjunctivitis. In contrast to temperature, outpatient admission was negatively correlated with humidity [20]. In addition, previous studies have shown that environmental factors are also involved in dispersing allergens. For example, low relative humidity promotes the airborne spread of pollen [21]. High temperatures can promote pollen dispersal, which is also associated with allergic conjunctivitis risk [22,23]. However, earlier research only evaluated the single impacts of either temperature or humidity, which may confound each other and fail to capture the overall effect of both. Therefore, evaluating the adverse impacts of temperature and humidity on child and adolescent allergic conjunctivitis in combination may be more practical.

The Humidex, an innovative and comprehensive index first proposed by Masterton and Richardson at the Meteorological Service of Canada, can consider both temperature and humidity, allowing a more comprehensive assessment of the association between temperature, humidity, and human comfort level [24]. The body temperature in dry air differs from that in humid air. The higher the temperature, the lower the comfort level and the greater the discomfort. Humidex provides a better understanding of the body's response to climate by effectively integrating temperature and humidity. Moreover, early studies discovered a link between humidex and depression, bacillary dysentery and asthma [25-27]. However, to our knowledge, the relationship between humidex and allergic conjunctivitis in children remains unexplored. Temperature and humidity are each independently linked to mental disorders. Given that humidex is a combination of variables that includes temperature and humidity, a thorough investigation is needed to identify the association between humidex and allergic conjunctivitis admissions to provide susceptible groups with effective intervention methods in a timely manner.

In addition to meteorological factors, air pollutants are a significant cause of allergic reactions, specifically asthma. In recent years, emerging research has shown that alterations in temperature and particulate matter less than 2.5 µm (μm) (PM_2.5_) have synergistic consequences for human wellness [28,29]. Mimura et al. also claimed that short-term exposure to PM_2.5_ was related to the development of allergic conjunctivitis [30]. For example, researchers from England found that the effect of short-term and long-term exposure to PM_2.5_ could raise the risk of respiratory disease admissions during periods of higher temperature variability, suggesting that temperature variability and air pollutants have a synergistic effect on health risk and that both meteorological factors and air pollutants need to be considered [29]. A study from China is consistent with this finding and found a similar synergistic effect of temperature variation and PM_2.5_ exposure on the risk of paediatric asthma hospital admissions [31]. However, the synergistic relationship of humidex and PM_2.5_ on admission for child and adolescent allergic conjunctivitis has not yet been studied.

In this study, we obtained large-scale data on outpatient visits for child and adolescent allergic conjunctivitis from multiple hospitals in Shanghai, China. We assessed the relationship between humidex and outpatient visits for child and adolescent allergic conjunctivitis utilising a distributed lag nonlinear model (DLNM). We also analysed how this relationship is modified by different subgroups (genders, ages and seasons) and the burden of allergic conjunctivitis attributable to humidex.

## METHODS

### Study region

Shanghai is located in East China (31°25′ N, 122°49′ E) on the western coastline of the Pacific Ocean and the eastern frontier of Asia and is part of the alluvial plain of the Yangtze River Delta. It is characterised by a subtropical monsoon climate and the river network mainly includes the Huangpu River, the main waterway flowing through the city, and its tributaries, the Suzhou River. The city of Shanghai is 6340.5 km^2^ (km^2^) in total size. As of the end of 2022, the resident population of Shanghai is 24.75 million.

### Data collection

This time-series research was carried out at the Eye, Ear, Nose and Throat Hospital of Fudan University and Children's Hospital of Fudan University from 1 January 2017 to 31 December 2022 under the authorisation of the ethics committees of the Eye, Ear, Nose, and Throat Hospital of Fudan University and Children's Hospital of Fudan University. They are the largest ophthalmology facility and paediatric facility in Shanghai, respectively. Information on outpatient visits for children and adolescents (0-18 years) with allergic conjunctivitis was obtained from the Hospital Information System. All participants were diagnosed through a review of their symptoms and eye examination by an experienced ophthalmologist. The diagnosis of allergic conjunctivitis was based on the ICD-10 classification of diseases (H10.101). Personal characteristics, including gender (girl, boy), age (0-1 year, 2-6 years, 7-18 years), address, date of visit, first visit, and revisit, were also collected. Only revisiting more than 30 days was included in the final analysis.

The daily meteorological data from 1 January 2017 to 31 December 2022 were derived from the Shanghai Environmental Protection Agency through a fixed meteorological monitoring station, including relative humidity (%), mean temperature (°C), wind speed (metre per second (m/s)), precipitation (millimetre per 24-hour (mm/24-hour)) and air pressure (hectopascal (hPa)). The daily meteorological factor levels were expressed as the average of those fixed meteorological monitoring stations.

We also collected daily data on 24-hour mean PM2.5, particulate matter less than 10 μm (PM_10_), nitrogen dioxide (NO_2_), carbon monoxide (CO), sulphur dioxide (SO_2_), and 8-hour mean ozone (O_3_) from fixed municipal air pollutant monitoring sites in Shanghai during the research period. The average of those fixed air pollution monitoring sites was used to represent the daily airborne pollution levels.

### Calculation of humidex

In brief, humidex was defined as a composite indicator based on a combination of temperature and humidity, referring to the formula proposed by the Meteorological Service of Canada. Humidex is dimensionless but can be expressed as a dry temperature in °C. The feeling at this time is similar to a dry temperature of 40°C if the temperature stays at 30°C and the calculated humidex is 40°C. At the same temperature and relative humidity, humidity tends to be higher than the United States heat index. The humidex is often interpreted according to the following scale: humidex less than 29°C (no discomfort), humidex between 30 and 39°C (some discomfort), humidex between 40 and 45°C (significant discomfort), humidex between 45 and 54°C (hazardous) and humidex above 54°C (the danger of heat stroke). The calculation formula used in this research was provided by CSGNetwork (http://www.csgnetwork.com/) and was as follows:



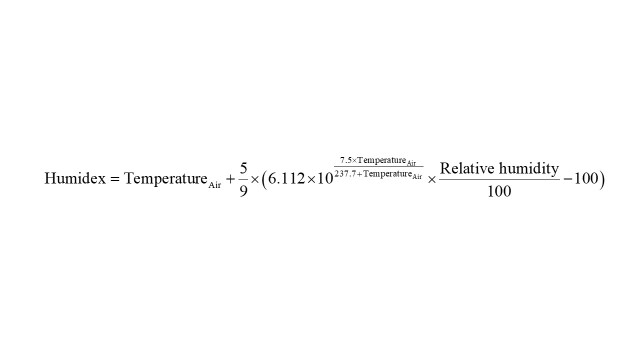



### Statistical analysis

Daily outpatient visits, the value of meteorological factors, and concentrations of six air pollutants were presented as an average value, standard deviation, minimum, 25th percentiles, 50th percentiles, 75th percentiles and maximum. The possible correlation between various variables was examined using Spearman's correlation.

To evaluate the exposure-response relationship between humidex and child and adolescent allergic conjunctivitis outpatient visits, we formed a time-series model. As the daily outpatient visit count for child and adolescent allergic conjunctivitis followed a Poisson distribution, the delayed effects of humidex on allergic conjunctivitis outpatient visits were investigated using a case-crossover approach based on a Poisson generalised linear model combined with DLNM [32]. Specifically, we establish exposure-response dimension fitting using the natural cubic spline (ns) function and exposure-lag dimension fitting. We also use the ns function to establish the cross-basis function of the fitted exposure-lag-response independent variable (humidex). In addition, considering that to demonstrate the lag effect over a more extended period, we set the maximum number of lag days to 14 days [26,33]. To control for confounding effects by environmental factors and air pollutants, we define the degrees of freedom (df) of the ns of environmental factors and air pollutants to three the df of the natural cubic spline of time as seven. Dummy variables for weeks and legal holidays are also set to control for the confounding effect of holidays and days of the week (DOW). Finally, to quantify the exposure-lag-response link between humidex and the admission of allergic conjunctivitis, we obtained the fundamental model shown as follows:

Y_t_ ~ Poisson(μ_t_)

Log(Y_t_) = α + βHumidex_t,l_ + ns(Mean temperature, df = 3)  + ns(Relative humidity, df = 3) + ns(Precipitation, df = 3)  + ns(Air pressure, df = 3) + ns(Wind speed, df = 3)  + ns(PM_2.5_, df = 3) + ns(PM_10_,df = 3) + ns(SO_2_, df = 3)  + ns(NO_2_, df = 3) + ns(CO, df = 3) + ns(O_3_, df = 3)  + ns(Time, df = 7 × 14) + γDOW_t_ + δHoliday_t_

In brief, t refers to the day during the exposure period; μ_t_ refers to the day_t_ counts of the child and adolescent allergic conjunctivitis; α refers to the intercept; β, γ, and δ refer to the coefficients of humidex, DOW, and holiday, respectively; l refers to the lag day (14 days); DOW is a dummy variable for weeks. Holiday refers to the Chinese public holiday, with 0 and 1 indicating non holidays and holidays, respectively. The effects of humidex on allergic conjunctivitis were assessed using relative risks (RRs) and 95% confidence intervals (CIs). We estimated the single-day lag effects of humidex during the specific lag day. For instance, the humidex on the patient's visitation day is lag 0, the humidex on the previous day is lag 1 and so on. For cumulative-day lag effects, we estimated the moving average lag effects of humidex. For example, the humidex on lag 0-1 was obtained by lag 0 + lag 1, and so on. To evaluate the suitability of the models and partial autocorrelation of residuals, residual plots and partial autocorrelation (PACF) were utilised. The df values are selected according to the minimum sum of the absolute values of PACF.

We also evaluated the exposure-response association between humidex and child and adolescent allergic conjunctivitis outpatient visits in different subgroups [34]. Subgroup analysis was conducted on gender (girl, boy), age (0-1 year, 2-6 years, 7-18 years) and season (warm season, cold season). The warm season lasts from April to September, and the cold season lasts from October to March of the next year [35]. The statistical significance of differences among subgroups was assessed using the formula below [34]:



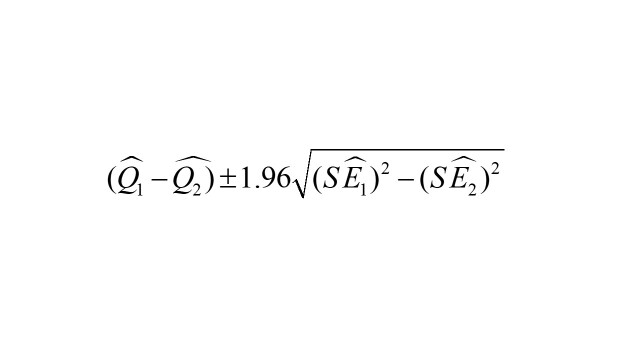



Where Q_1_ and Q_2_ refer to the estimates from those subgroups SE_1_ and SE_2_ refer to the standard errors.

Furthermore, to verify the disease burden of child and adolescent allergic conjunctivitis, we estimated the attributable fraction (AF) and attributable number (AN) of cases of allergic conjunctivitis with humidex cumulative exposure at various lag day_t_. The calculation formula for AF and AN is as follows [36]:

AF_t_ = 1 – exp( – βt)

An_t_ = Af_t_ x N_t_

where β_t_ refers to the relative risks at day_t_; N_t_ refers to the counts of child and adolescent allergic conjunctivitis at day_t_.

To determine the robustness of our model, sensitivity studies were carried out: (1) the df of meteorological factors and air pollutants were changed to df = 3-5; (2) the df of time was changed to df = 7-9; and (3) referring to the previous study, the lag periods were extended to 21 days (three weeks) [37].

All analyses were performed in R software (4.2.2) by using the “dlnm”, “splines”, “mgcv”, “tsModel”, “nlme”, “reshape2”, and “ggplot2” R packages. *P* < 0.05 refers to statistical significance (two-tailed).

## RESULTS

### Descriptive analysis

**Table 1** describes the daily information on outpatient visit for children and adolescents with allergic conjunctivitis from 1 January 2017 to 31 December 2022. Our cohort contained 147 090 cases altogether, and the daily mean and standard deviation (SD) were 67.13 and 43.41, respectively. There were 50 820 outpatient visits by girls for allergic conjunctivitis, almost half of the boys (96 263 cases). In the subgroup of age, the highest group was 2-6 years (preschool-aged) with 59 326 cases, followed by 7-18 years (school-aged group) with 81 334 cases and the smallest group was 0-1 year (infants). Moreover, 87 302 and 59 788 cases were identified during the warm and cold seasons, respectively. **Table 2** presents the descriptive data of humidex, meteorological factors, and air pollutants. During this period, the daily mean humidex ranged from -8 to 55. The number of heat stress days (humidex >40) was 374 days, 17.07% of the total number of days (Table S1 in the **Online Supplementary Document**). In addition, the daily average mean temperature, relative humidity, wind speed, air pressure, and precipitation were 18.57°C, 76.65%, 3.70 m per second (m/s), 1016.07 hPa, and 3.69 mm/24 hours, respectively. The 24-hour average air pollution concentrations of PM_2.5_, PM_10_, SO_2_, NO_2_, and CO and the 8-hour average of O_3_ were 31.65 μg/m^3^, 45.67 μg/m^3^, 7.36 μg/m^3^, 36.94 μg/m^3^, 0.67 μg/m^3^ and 86.37 μg/m^3^.

**Table 1:** Description summary of daily outpatient visits of children and adolescents with allergic conjunctivitis in Shanghai, 2017-2022

Variables	Sum	Mean	SD	Minimum	P_25_	Median	P_75_	Maximum
**Daily outpatient visit**s	147 090	67.13	43.41	0	26	69	99	202
**Child's sex**								
Girl	50 827	23.20	15.26	0	9	24	34	71
Boy	96 263	43.94	28.99	0	17	45	65	142
**Age (in years)**								
0-1	6430	37.12	24.09	0	12	40	54	120
2-6	81 334	2.93	3.10	0	0	2	4	20
7-18	59 326	27.08	21.02	0	10	22	41	115
**Season**								
Warm season (April to September)	87 302	79.51	45.13	0	49	85.5	110	202
Cold season (October to March)	59 788	54.70	37.76	0	15	58	81	156

SD – standard deviation, P_25_ – 25th percentile, P_75_ – 75th percentile

**Table 2:** Description summary of daily air pollutant concentration, humidex, and meteorological factors in Shanghai, 2017-2022

Variables	Mean	SD	Minimum	P_25_	Median	P_75_	Maximum
**Air pollutant concentration (24-h average)**							
PM_2.5_ (μg/m^3^)	31.65	21.41	0	16	26	40	191
PM_10_ (μg/m^3^)	45.67	25.59	0	29	39	57	308
SO_2_ (μg/m^3^)	7.36	3.51	3	5	6	9	31
NO_2_ (μg/m^3^)	36.94	17.64	4	24	34	46	118
CO (μg/m^3^)	0.67	0.21	0.30	0.51	0.61	0.79	1.80
**Air pollutant concentration (8-h average)**							
O_3_ (μg/m^3^)	86.37	41.02	8	58	79	108	295
**Meteorological factors**							
Mean temperature (°C)	18.57	8.74	-4.50	11	19	26	35.50
Relative humidity (%)	76.65	10.32	39	70	78	84	97
Humidex (°C)	23.47	14.36	-8	11	22	35	55
Wind speed (m/s)	3.70	1.43	1	3	4	4	14
Air pressure (hPa)	1016.07	9.08	987	1008	1017	1023	1039
Precipitation (mm/24 h)	3.69	8.94	0	0.01	0.26	3.17	117.86

SD – standard deviation, P_25_ – 25^th^ percentile, P_75_ – 75^th^ percentile, h – hour, PM_2.5_ – particulate matter less than 2.5 µm, μg – microgramme, m^3^ – cubic metre, PM_10_ – particulate matter less than 10 μm, SO_2_ – sulphur dioxide, NO_2_ – nitrogen dioxide, CO – carbon monoxide, O_3_ – ozone, m – metre, s – second, hPa – hectopascal, mm – millimetre

**Figure 1:**
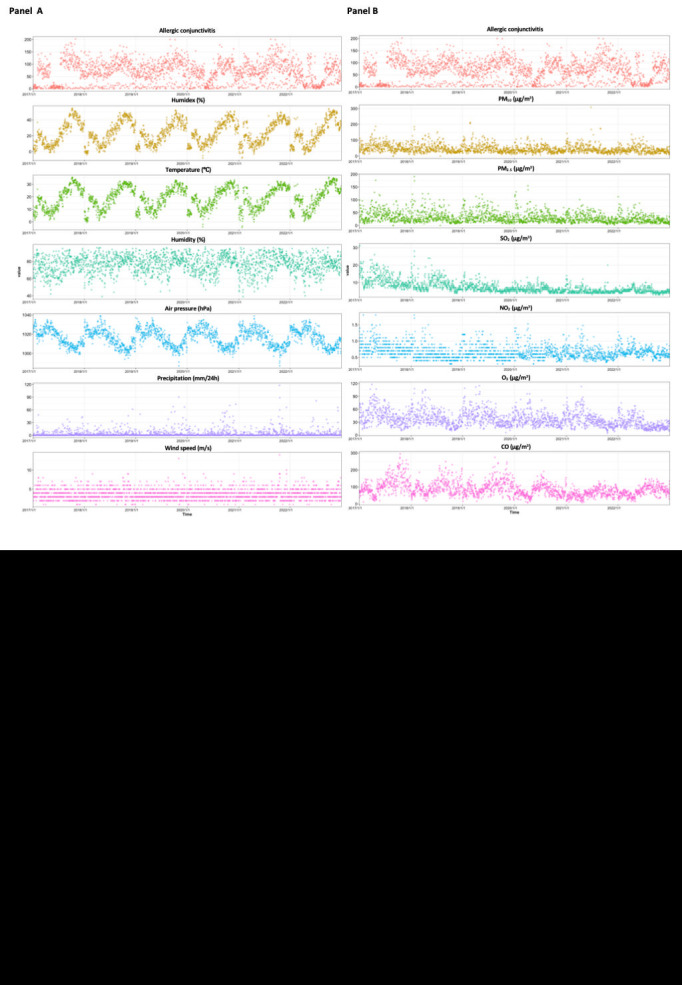
Daily changes in child and adolescent allergic conjunctivitis outpatient visits, humidex, meteorological factors, and air pollutants in Shanghai, 2017-2022.

**Figure 2:**
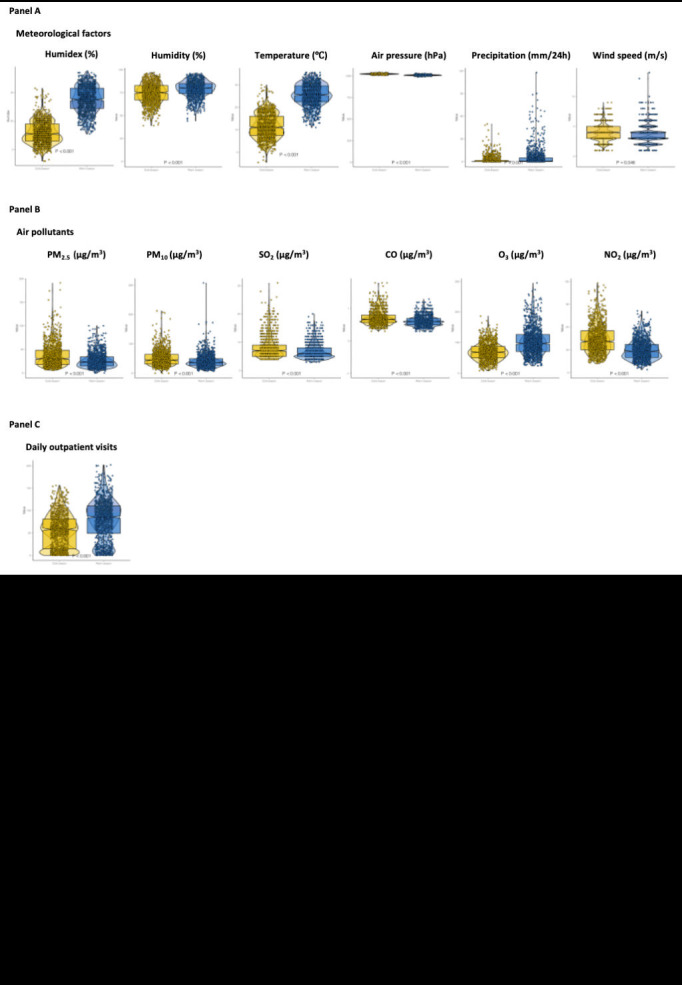
Violin and boxplot showing the variation in humidex, meteorological factors, air pollutants, and child and adolescent allergic conjunctivitis outpatient visits in warm and cold seasons from 2017 to 2022. **Panel A**. The distribution mode of humidex and meteorological factors between warm and cold seasons. **Panel B.** The distribution mode of six air pollutants between warm and cold seasons. **Panel C.** The distribution mode of child and adolescent allergic conjunctivitis outpatient visits between warm and cold seasons.

**Figure 1**, panels A and B display daily time-series distributions of humidex, meteorological factors, air pollutants, and outpatient visits for allergic conjunctivitis. Seasonal and periodic trends were observed in the distribution of the four meteorological parameters and the two air pollutants, as well as in the total number of daily outpatient visits for allergic conjunctivitis. Among them, the mean temperature exhibited the same pattern as the humidex, which was reduced in winter and more elevated during the summer. The pattern of changes in air pressure is the opposite of this. The remaining meteorological factors and air pollutants fluctuated widely. The change in meteorological parameters and air pollution in the warm and cold seasons is depicted by the violin and boxplot (**Figure 2**, panels A and B). PM_2.5_, PM_10_, SO_2_, NO_2_, and CO concentrations increased in the warm season, while O_3_ increased in the cold season. In addition, the humidex, mean temperature, relative humidity, wind speed and precipitation was higher in the warm season. The air pressure was higher in the cold season. Additionally, compared to the cold season, the number of cases of daily allergic conjunctivitis was higher in the warm season (**Figure 2**, panel C).

Using Spearman's method, we additionally evaluated the relationship between climate variables and air pollution (Figure S1 in the **Online Supplementary Document**). The highest correlation was found between air pressure and humidex. Since both temperature and humidity show a negative correlation with air pressure, the index that makes up the two also shows a negative correlation with air pressure. The details of the coefficients are shown in **Table 3**.

**Table 3:** Spearman’s correlation coefficients of daily humidex, meteorological factors, and air pollutants in Shanghai, 2017-2022

Variables	PM_2.5_	PM_10_	SO_2_	CO	NO_2_	O_3_	Humidity	Air pressure	Precipitation	Wind speed	Humidex	Temperature
PM_2.5_	1.00*											
PM_10_	0.82*	1.00*										
SO_2_	0.53*	0.62*	1.00*									
CO	0.74*	0.59*	0.44*	1.00*								
NO_2_	0.68*	0.61*	0.53*	0.60*	1.00*							
O_3_	0.17*	0.23*	0.31*	-0.01	-0.13*	1.00*						
Humidity	-0.14*	-0.45*	-0.36*	-0.07*	-0.13*	-0.21*	1.00*					
Air pressure	0.19*	0.27*	0.23*	0.19*	0.39*	-0.31*	-0.46*	1.00*				
Precipitation	-0.25*	-0.48*	-0.37*	-0.09*	-0.23*	-0.18*	0.74*	-0.37*	1.00*			
Wind speed	-0.34*	-0.23*	-0.13*	-0.32*	-0.49*	-0.06†	-0.02	0.02	0.12‡	1.00*		
Humidex	-0.31*	-0.31*	-0.25*	-0.30*	-0.46*	0.34‡	0.31*	-0.86*	0.24*	-0.06†	1.00*	
Temperature	-0.31*	-0.28*	-0.23*	-0.30*	-0.46*	0.36*	0.24*	-0.84*	0.19*	-0.06†	1.00*	1.00*

PM_2.5_ – particulate matter less than 2.5 µm, PM_10_ – particulate matter less than 10 μm, SO_2_ – sulphur dioxide, CO – carbon monoxide, NO_2_ – nitrogen dioxide, O_3_ – ozone

*P < 0.001.

†*P* < 0.01.

### Lag-response analysis between humidex and allergic conjunctivitis

**Figure 3:**
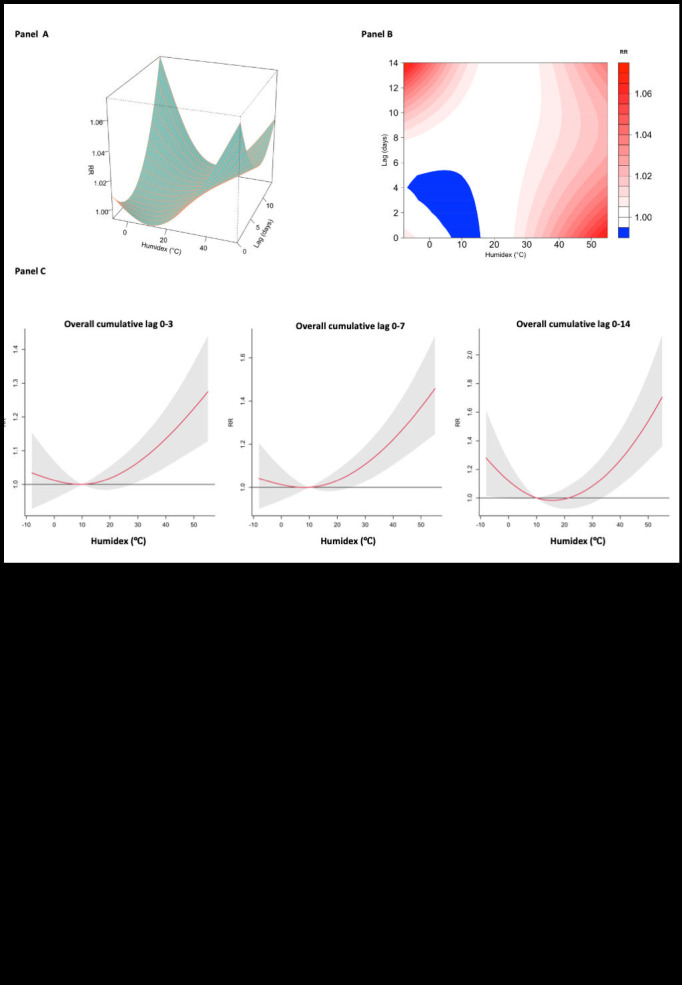
The association of humidex in the relative risk of child and adolescent allergic conjunctivitis outpatient visits. **Panel A.** The 3D illustration showing the correlation between humidex and the daily admission of allergic conjunctivitis cases at various humidex levels and lag days. **Panel B.** Contour plot showing the exposure-lag-response relationship between humidex and allergic conjunctivitis incidence on various lag days. Blue areas indicate relative risks (RR)<1; red areas indicate relative risks (RR)>1. **Panel C.** Overall cumulative lag effect of the increase in humidex on allergic conjunctivitis outpatient visits at lag 0-3, lag 0-7, and lag 0-14.

**Figure 3**, panels A and B present the exposure-lag-response effect of humidex associated with hospitalisation for children and adolescents with allergic conjunctivitis at various lag days. Hospitalisations for allergic conjunctivitis were significantly inversely correlated with humidex exposure in a nonlinear pattern. In addition, we found a significant overall cumulative lag effect of humidex on outpatient visits for allergic conjunctivitis at lag 0-3, 0-7, and 0-14 (**Figure 3**, panel C).

**Figure 4:**
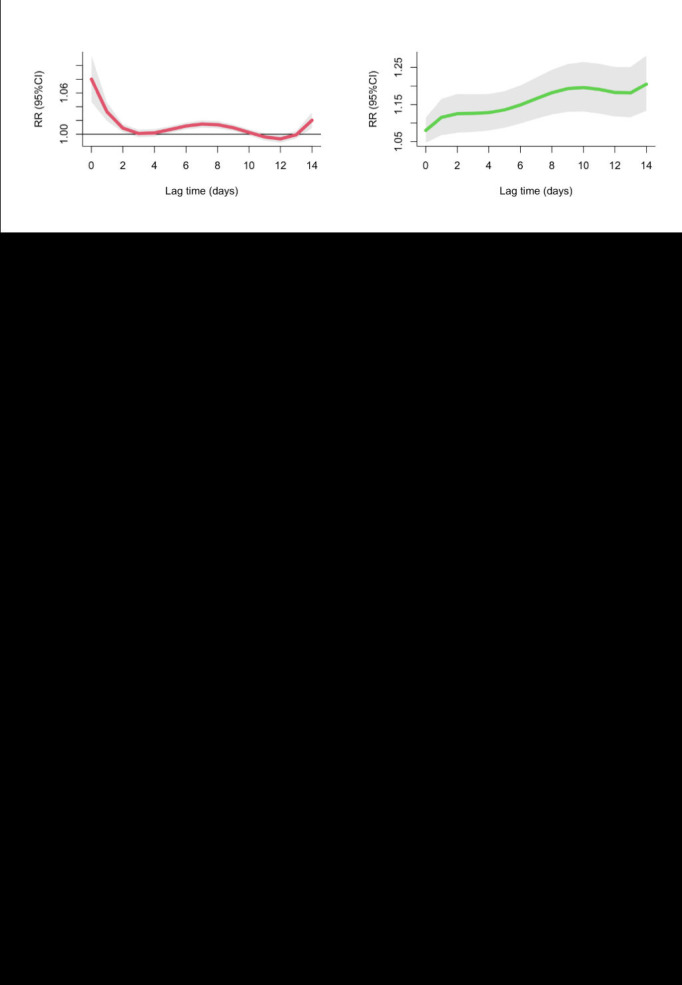
The association of humidex in the relative risk of child and adolescent allergic conjunctivitis outpatient visits. **Panel A.** The single-day lag pattern. **Panel B.** The cumulative-lag effect pattern. Grey areas indicate 95% confidence interval (CI).

**Figure 4** displays the association of humidex with the RR of child and adolescent allergic conjunctivitis admission at different lag days. In the single-day lag pattern, these results demonstrated a substantial correlation between higher humidex exposure and allergic conjunctivitis at lag 0 (RR = 1.08, 95% CI = 1.05-1.11) to lag 2 (RR = 1.01, 95% CI = 1.00-1.01), lag 5 (RR = 1.01, 95% CI = 1.00-1.01) to lag 9 (RR = 1.01, 95% CI = 1.00-1.01), and lag 14 (RR = 1.02, 95% CI = 1.01-1.03), with the peak RR at lag 14 (Table S2 in the **Online Supplementary Document**). These results demonstrated that higher humidex exposure was substantially responsible for allergic conjunctivitis based on the cumulative-lag effect pattern at lag 0-0 (RR = 1.08, 95% CI = 1.05-1.11) to lag 0-14 (RR = 1.21, 95% CI = 1.13-1.28), with the peak RR at lag 14 (Table S2 in the **Online Supplementary Document**).

### Subgroup analysis

**Figure 5:**
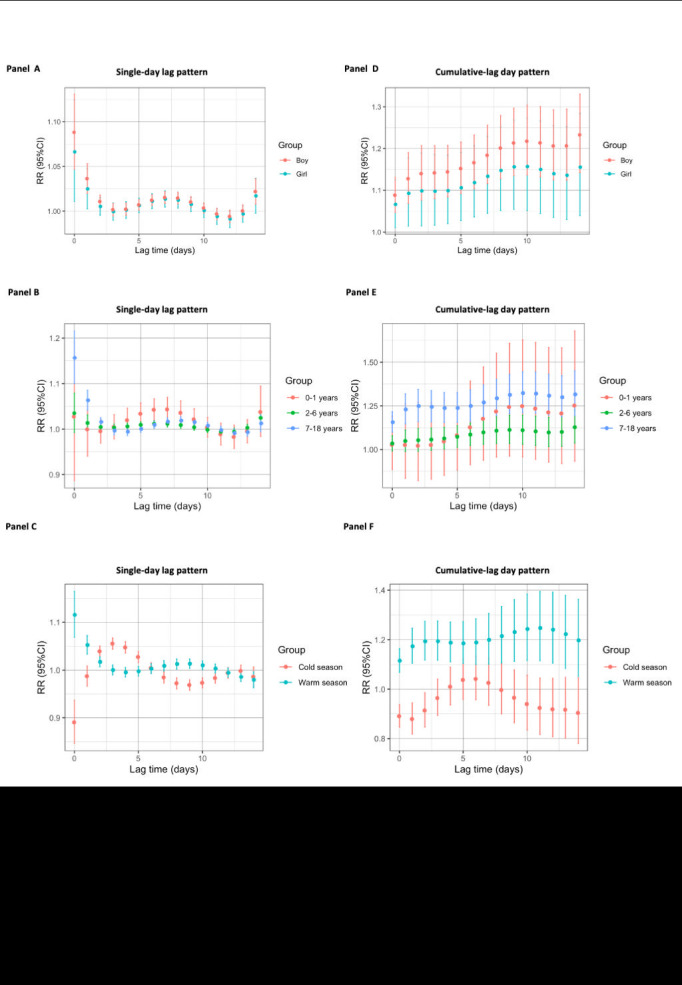
The relationship between humidex and the relative risk of outpatient visits for allergic conjunctivitis in children and adolescents, categorised by child's gender, age, and season, over various lag days. The single-day lag pattern (**Panel A**) and cumulative-lag effect pattern (**Panel B**) in girls and boys. The single-day lag pattern (**Panel C**) and cumulative-lag effect pattern (**Panel D**) in 0-1 year, 2-6 years, and 7-18 years. The single-day lag pattern (**Panel E**) and cumulative lag effect pattern (**Panel F**) in the warm season and cold seasons.

We carried out a stratified analysis to find sensitive subgroups based on sex, age and season. The results of gender subgroup analysis indicated that there was a substantial link between humidex exposure and the risk of allergic conjunctivitis in boys, 7-18 years, and warm season subgroups in a single-day lag pattern (**Figure 5**, panels A to C, Table S3-S5 in the **Online Supplementary Document**). In the boy subgroup, a significant RR of allergic conjunctivitis was found at lag 0 (RR = 1.09, 95% CI = 1.05-1.13) to lag 2 (RR = 1.01, 95% CI = 1.00-1.02), lag 5 (RR = 1.01, 95% CI = 1.00-1.01) to lag 9 (RR = 1.01, 95% CI = 1.00-1.02), and lag 14 (RR = 1.02, 95% CI = 1.01-1.04), with the peak RR at lag 0. In the girl subgroup, a significant RR of allergic conjunctivitis was found at lag 0 (RR = 1.07, 95% CI = 1.05-1.12) to lag 1 (RR = 1.02, 95% CI = 1.00-1.05) and lag 6 (RR = 1.01, 95% CI = 1.00-1.02) to lag 8 (RR = 1.01, 95% CI = 1.00-1.02), with the peak RR at lag 0. The maximum RR was lower than that in the boy subgroup. Moreover, in the 0-1 year subgroup, a significant RR for allergic conjunctivitis was found at lag 5 (RR = 1.03, 95% CI = 1.01-1.06) to lag 8 (RR = 1.04, 95% CI = 1.01-1.06), with the peak RR at lag 7 (RR = 1.04, 95% CI = 1.01-1.06). The 7-18 years subgroup had a stronger association at lag 0 (RR = 1.16, 95% CI = 1.10-1.22) to lag 2 (RR = 1.02, 95% CI = 1.01-1.03) and lag 6 (RR = 1.01, 95% CI = 1.00-1.02) to lag 10 (RR = 1.01, 95% CI = 1.00-1.01), with the peak RR at lag 0. Furthermore, in the warm season subgroup, the relationship between humidex and allergic conjunctivitis was positive at lag 0 (RR = 1.12, 95% CI = 1.07-1.16) to lag 2 (RR = 1.02, 95% CI = 1.01-1.03) and lag 8 (RR = 1.01, 95% CI = 1.00-1.02) to lag 10 (RR = 1.01, 95% CI = 1.00-1.02), with the peak RR at lag 0. In the cold season subgroup, the relationship between humidex and allergic conjunctivitis was positive at lag 2 (RR = 1.04, 95% CI = 1.03-1.05) to lag 5 (RR = 1.03, 95% CI = 1.02-1.04), with the peak RR at lag 3 (RR = 1.06, 95% CI = 1.04-1.07).

For cumulative risk, a similar tendency was also observed in the gender subgroup. The RR in the boy subgroup (RR = 1.23, 95% CI = 1.14-1.33) was higher than that in the girl group (RR = 1.16, 95% CI = 1.04-1.28) at lag 0-14 (**Figure 5**, panel D). In addition, in the age subgroups, humidex was significantly associated with allergic conjunctivitis in the 7-18-year subgroup at lag 0-0 (RR = 1.16, 95% CI = 1.10-1.22) to lag 0-14 (RR = 1.32, 95% CI = 1.19-1.45). The 7-18-year subgroup was more sensitive than the 2-6-year subgroup. However, there was no significant association in the 0-1 year subgroup (**Figure 5**, panel E). However, in the seasonal subgroups, the relationship between warm season and cold season medium humidex exposure and the cumulative RR of allergic conjunctivitis was the opposite. Humidex was related to the cumulative RR of allergic conjunctivitis in the warm season, with the peak RR (RR = 1.20, 95% CI = 1.05-1.36) at lag 0-14 (**Figure 5**, panel F).

In both the overall population and various subgroups, **Table 4** displays the cumulative lag effect of exposure to extremely low humidex (1st) and extremely high humidex (99th) on hospitalisations for allergic conjunctivitis. Extremely low humidex had no obvious correlation with allergic conjunctivitis in the total, child's sex, and age subgroups, while it showed an apparent association in the cold season at ag 0-1, lag 0-3 and lag 0-14. In addition, we discovered that highly high humidex showed higher risk in total at lag 0-1, lag 0-3, lag 0-7, and lag 0-14. A similar trend was also presented in both the boy and girl subgroups, the 7-18-year subgroup, and the warm season subgroup, whereas the relationship between infants and the cold season was insignificant.

**Table 4:** Extremely low and high humidex on the cumulative relative risk of allergic conjunctivitis over different lag days stratified by child's gender, age and season

Groups	Extremely low humidex (1^st^)				Extremely high humidex (99^th^)			
	**Lag 0-1**	**Lag 0-3**	**Lag 0-7**	**Lag 0-14**	**Lag 0-1**	**Lag 0-3**	**Lag 0-7**	**Lag 0-14**
**Total**	0.99 (0.98-0.99)	0.99 (0.98-0.99)	0.98 (0.98-0.99)	0.98 (0.98-0.99)	1.74 (1.39-2.18)	1.83 (1.45-2.31)	2.19 (1.71-2.79)	2.59 (1.89-3.55)
**Child's gender**								
Girl	0.99 (0.98-1.00)	0.99 (0.98-1.00)	0.99 (0.98-1.00)	0.99 (0.98-1.00)	1.57 (1.07-2.30)	1.61 (1.09-2.38)	1.89 (1.25-2.87)	2.09 (1.22-3.58)
Boy	0.99 (0.98-0.99)	0.99 (0.98-0.99)	0.98 (0.98-0.99)	0.98 (0.97-0.99)	1.85 (1.40-2.43)	1.96 (1.48-2.61)	2.36 (1.75-3.19)	2.90 (1.97-4.29)
**Age (in years)**								
0-1	1.00 (0.98-1.02)	1.00 (0.98-1.02)	0.98 (0.96-1.01)	0.98 (0.95-1.01)	1.14 (0.40-3.27)	1.14 (0.39-3.37)	2.29 (0.72-7.22)	3.15 (0.70-14.11)
2-6	1.00 (0.99-1.00)	0.99 (0.99-1.00)	0.99 (0.98-1.00)	0.99 (0.98-1.00)	1.28 (0.95-1.72)	1.33 (0.98-1.81)	1.61 (1.16-2.23)	1.85 (1.21-2.83)
7-18	0.98 (0.97-0.99)	0.98 (0.97-0.99)	0.98 (0.97-0.98)	0.97 (0.96-0.98)	2.87 (2.01-4.10)	3.05 (2.12-4.41)	3.38 (2.30-4.97)	4.06 (2.47-6.67)
**Season**								
Warm	0.98 (0.98-0.99)	0.98 (0.98-0.99)	0.98 (0.97-0.99)	0.98 (0.97-0.99)	2.26 (1.66-3.08)	2.47 (1.76-3.46)	2.53 (1.64-3.90)	2.51 (1.30-4.84)
Cold	1.01 (1.01-1.02)	1.00 (1.00-1.01)	1.00 (0.99-1.01)	1.01 (1.00-1.03)	0.52 (0.36-0.75)	0.83 (0.56-1.22)	1.14 (0.71-1.82)	0.60 (0.28-1.26)

### Attributable fraction and attributable number analysis

**Table 5** presents the AF and AN of humidex in different subgroups for child and adolescent allergic conjunctivitis. We chose four time points (lag 0-3, lag 0-7, and lag 0-14) to compare the correlation coefficients. Among these four time points, the AF of allergic conjunctivitis was 0.11 (95% CI = 0.07-0.15), 0.14 (95% CI = 0.10-0.18) and 0.17 (95% CI = 0.12-0.22) at lag 0-3, lag 0-7 and lag 0-14, respectively. Moreover, the AN of allergic conjunctivitis cases was 16 451 (95% CI = 10 413-22 222), 20 912 (95% CI = 14 759-26 780), and 25 026 (95% CI = 17 235-32 349) at lag 0-3, lag 0-7, and lag 0-14, respectively. For the child's gender subgroup, the boy had the highest AF (0.19, 95% CI = 0.12-0.25) and AN (18 164, 95% CI = 11 969-23 903) at lag 0-14. Similar to these results, the 0-18 years subgroup also had the highest AF (0.24, 95% CI = 0.16-0.31) and AN (14 244, 95% CI = 9629-18 430) at lag 0-14. In addition, the warm season subgroup had the highest AF (0.17, 95% CI = 0.9-0.23) and AN (9945, 95% CI = 5516-14 013) at lag 0-7.

**Table 5:** Attributable fractions and attributable numbers with 95% confidence interval (CI) of daily admission for allergic conjunctivitis over different lag days stratified by child's gender, age and season

Groups	Total case, n	Attributable fractions, % (95% CI)			Attributable numbers, n (95% CI)		
**Lag 0-3**	**Lag 0-7**	**Lag 0-14**	**Lag 0-3**	**Lag 0-7**	**Lag 0-14**
**Total**	147 090	0.11 (0.07to 0.15)	0.14 (0.10 to 0.18)	0.17 (0.12 to 0.22)	16 451 (10 413 to 22 222)	20 912 (14 759 to 26 780)	25 026 (17 235-32 349)
**Child's gender**							
Girl	50 827	0.09 (0.02 to 0.16)	0.12 (0.04 to 0.19)	0.13 (0.04 to 0.22)	4525 (816 to 7958)	5983 (2190 to 9480)	6831 (1928-11 242)
Boy	96 263	0.12 (0.07 to 0.17)	0.16 (0.10 to 0.20)	0.19 (0.12 to 0.25)	11 940 (7102 to 16 515)	14 937 (10 012 to 19 580)	18 164 (11 969-23 903)
**Age (in years)**							
0-1	6430	0.03 (-0.21 to 0.21)	0.15 (-0.07 to 0.32)	0.20 (-0.07 to 0.40)	165 (-1318 to 1364)	962 (-420 to 2066)	1294 (-464-2603)
2-6	81 334	0.05 (0.00 to 0.11)	0.09 (0.03 to 0.15)	0.11 (0.04 to 0.18)	4391 (-403 to 8904)	7268 (2374 to 11860)	9227 (2947-15004)
7-18	59 326	0.20 (0.14to 0.25)	0.21 (0.15 to 0.27)	0.24 (0.16 to 0.31)	11 666 (8106 to 14 979)	12 608 (8947 to 16 003)	14 244 (9629-18 430)
**Season**							
Warm	87 302	0.16 (0.11 to 0.22)	0.17 (0.09 to 0.23)	0.16 (0.05 to 0.27)	9713 (6284to 12 923)	9945 (5516 to14 013)	9860 (2986-15 903)
Cold	59 788	-0.04 (-0.12 to 0.04)	0.02 (-0.07 to 0.11)	-0.11 (-0.28 to 0.04)	-3256 (-10 399 to 3365)	2154 (-6126 to 9701)	-9295 (-24 518-3855)

CI – confidence interval

### Sensitivity assessment

In this sensitivity assessment, we changed the parameter settings of time (df = 7-8), meteorological factors (df = 3-5), and air pollutants (df = 3-5) and our model remained stable (Figures S2 and S3 in the **Online Supplementary Document**). Additionally, the major outcomes under the exposure-lag pattern were not significantly altered when the maximum lag days were increased from 14 to 21 (Figure S4 in the **Online Supplementary Document**). According to the above results, the model used in this study was robust.

### Synergistic effect of humidex on PM_2.5_

**Figure 6:**
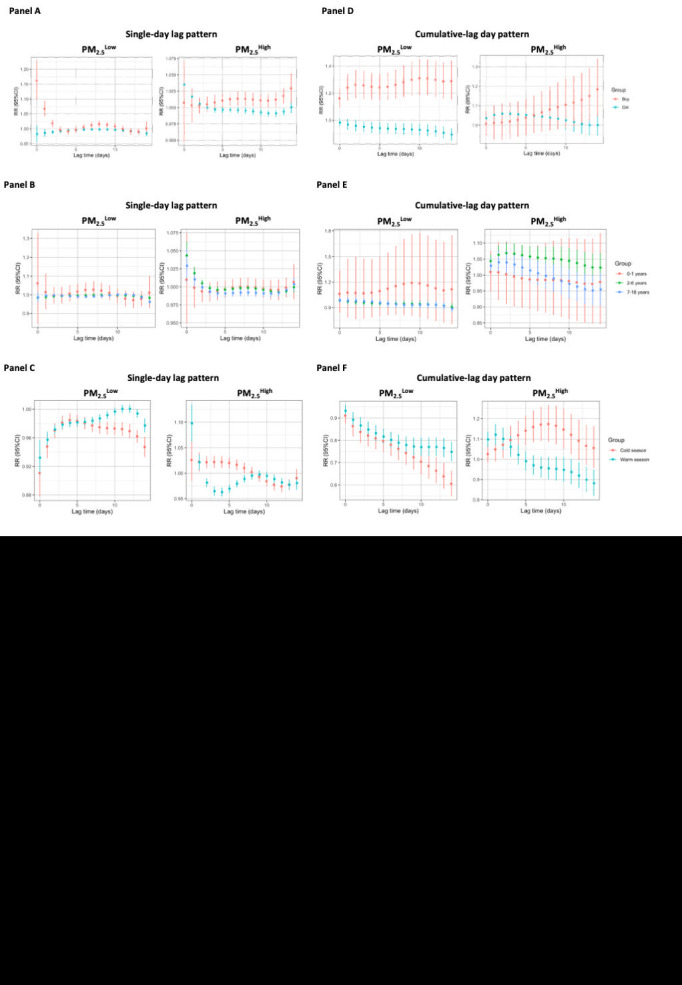
The relationship between humidex and the relative risk of outpatient visits for allergic conjunctivitis in children and adolescents, categorised by child's gender, age, and season, over various lag days in the PM_2.5_^Low^ and PM_2.5_^High^ categories. The single-day lag pattern (**Panel A**) and cumulative-lag effect pattern (**Panel B**) in girls and boys. The single-day lag pattern (**Panel C**) and cumulative-lag effect pattern (**Panel D**) in 0-1 year, 2-6 years, and 7-18 years. The single-day lag pattern (**Panel E**) and cumulative lag effect pattern (**Panel F**) in the warm season and cold seasons.

In this modification assessment, we divided the subgroups into two categories (PM_2.5_^Low^ and PM_2.5_^High^) based on the median concentration of PM_2.5_. **Figure 6** shows the RR of humidex exposure on outpatient visits for allergic conjunctivitis in gender, aged and seasonal subgroups in the PM_2.5_^Low^ and PM_2.5_^High^ categories within a two-week lag period. Based on the single-day lag pattern, we found that the relationship between humidex exposure and allergic conjunctivitis was significant in the boy and girl subgroups in the PM_2.5_^Low^ and PM_2.5_^High^ categories. In the PM_2.5_^Low^ category, the boy subgroup had the highest RR at lag 0 (**Figure 6**, panel A). There was no significant correlation in the age subgroup (**Figure 6**, panel B). For the seasonal subgroup, the effects of humidex exposure on outpatient visits for allergic conjunctivitis were significant in the PM_2.5_^High^ category in the warm season. The peak RR was 1.10 at lag 0 (**Figure 6**, panel C). For PM_2.5_^High^, the effects of humidex exposure on hospitalisations for allergic conjunctivitis were only significant in the boy subgroup in the cumulative-lag day pattern (**Figure 6**, panel D). This study is the first to examine how humidex affects outpatient visits for allergic conjunctivitis and whether there are any differences in how humidifiers affect different subgroups of patients. It is important to note that in PM_2.5_^Low^ compared to PM_2.5_^High^, the RR of the boy subgroup was greater. There were no significant differences for age subgroups in the PM_2.5_^Low^ and PM_2.5_^High^ categories (**Figure 6**, panel E). For PM_2.5_^High^, humidex exposure had a more detrimental impact on allergic conjunctivitis outpatient visits in the warm season than in the cold season (**Figure 6**, panel F).

## DISCUSSION

The frequency of allergic conjunctivitis is especially worrisome in children and adolescents and is becoming one of the most widespread ocular illnesses among children [38]. This study is the first to investigate the impact of humidex on child and adolescent allergic conjunctivitis outpatient visits, as well as whether the effect of humidifiers on allergic conjunctivitis varied among subgroups. Using 147 090 cases of allergic conjunctivitis in children and adolescents between January 2017 and December 2022, our results stated that humidex exposure increased the risk of child and adolescent allergic conjunctivitis after controlling for air pollutants and meteorological factors, including PM_2.5_, PM_10_, NO_2_, SO_2_, CO, O_3_, wind speed, temperature, humidity, precipitation, and air pressure. Furthermore, the effects of humidex were more prominent in boys, 7-18 years, and warm seasons in the cumulative-day lag model. Our findings also suggested that the highest AF and AN of humidex for allergic conjunctivitis were lag 0-14 at 0.17% (0.12-0.22%) and 25 026 (17 235-32 349), respectively. Overall, the findings of our study offer a thorough understanding of humidex's impact on allergic conjunctivitis outpatient visits and support the necessity for self-protection or self-management for the majority of allergic conjunctivitis patients.

The humidex is an index, as its name suggests. The objective is to gauge a person's comfort level and the temperature they are feeling given the current temperature and humidity. Additionally, humidity can affect the temperature [39]. The relationship between humidex exposure and disease outcomes has been investigated in several studies. For instance, in the province of Guangdong, the RR of outpatient visit for hand, foot and mouth disease (HFMD) was associated with exceptionally high humidex exposure [40]. In addition, a time-series study across multiple Chinese cities showed that an increase in humidex was linked to the risk of bacillary dysentery (BD), and the maximum RR was 1.45 (95% CI = 1.29-1.63) at 40.94 for humidex [27]. Additionally, epidemiological research has revealed that high humidex might dramatically increase the RR of depression [26]. Previous studies in China have shown that humidex increased the risk of childhood asthma, with a maximum cumulative RR of 1.356 (1.130-1.629) at lag 0-7 [25]. An epidemiological study indicated that low temperatures and humid conditions could cause acute asthma attacks [41]. However, another epidemiological study showed that the RR of acute asthma increased significantly in high-intensity exercise in low relative humidity and temperature environments. A possible biological explanation linking this phenomenon was exercise-induced bronchoconstriction due to hypothermia and humidity [42,43]. Additionally, because they are still growing and developing, children are more vulnerable to atmospheric exposure factors from the outside world and are more vulnerable to sustaining injuries. This is because their immune systems and physiological organs are still developing. To date, the underlying pathogenic processes have not been fully clarified. Therefore, more study is required to offer this link a thin thread of biological support.

Stratification analysis data showed that in the boy subgroup, exposure to high humidex caused a much greater RR of outpatient visits for allergic conjunctivitis than in the girl subgroup, possibly due to boys being more susceptible to allergic diseases, according to previous epidemiological studies [44]. In addition, in the age-specific subgroup analysis, compared with 0-1 year and 2-6 years, the cumulative-lag RR of allergic conjunctivitis was higher in 7-18 years. One possible explanation is that children who are in school may spend more time outside, have greater access to the outdoor environment and are more active than preschool-aged children, which leads to susceptibility in high-humidex environments. Preschool-aged children spend less time outdoors and mainly stay indoors. Additionally, earlier research discovered that breastfeeding contributes to decreased asthma [45]. Few other studies have reported the effects of humidex at different ages on allergic conjunctivitis outpatient visit in children. However, our findings are consistent with some research on asthma hospitalisation and humidex exposure in school-aged children compared with other age subgroups; humidex exposure is associated with a higher risk of asthma hospitalisation in school-aged children [25]. Seasons were also an influencing factor. In the seasonal stratification analysis, the number of admissions due to exposure to humidex was significantly higher in the warm season than in the cold season. The potential explanation was that the high humidity and high-temperature environment in the warm season might lead to higher humidex and cause a risk of allergic conjunctivitis.

In developing countries, the accelerated pace of anthropogenic climate change, such as urbanisation and industrialisation, impacts vulnerable populations, especially children and adolescents. Therefore, climatic factors should be combined with known clinical treatments when managing patients with allergic conjunctivitis. A previous study indicated that to mitigate the risk of allergic conjunctivitis from environmental factors, public health, environmental, or climate governance policies are needed [19,20,46], as well as education to understand the humidex and to reduce outdoor activities and drink more water during high humidex. In addition, protection and management of the ecosystem, increased awareness and response to the climate change crisis, increased investment in biotechnology solutions, and a shift to clean and renewable energy resources, such as tidal, wind, hydroelectric, and solar power [47,48]. However, to establish environmental risk factor control strategies suitable to regional climatic characteristics and sociodemographic conditions, more geodemographic-specific studies utilising humidex are required to track the current climate change risks' effect on paediatric allergies conjunctivitis.

Although the underlying biological mechanisms by which humidex affects paediatric allergic conjunctivitis have not been elucidated, several plausible pathophysiologic explanations may exist. A growing number of studies imply that low humidity aggravates the symptoms of patients with atopic dermatitis [49,50]. Additionally, experimental research indicated that higher environmental humidity alleviates the symptoms of atopic dermatitis-like mice by regulating CLCA2 expression in keratinocytes [51]. However, high environmental humidity (exceeding 80%) leads to dysregulation of the relevant metabolic pathways and exacerbates asthma symptoms in asthmatic mice [52]. High humidity exposure can also cause alterations in the structure and function of the gut microbial community, which may also be involved in the development of asthma [52,53]. A clinical observational study indicated that conjunctival surface temperature was related to the severity of conjunctival allergic symptoms [54]. As a result, local hypothermia can significantly relieve signs and symptoms of allergic conjunctivitis [55]. High temperatures lead to spatial and temporal patterns of vegetation that alter the pollen season and extend the flowering time of plants, which raises the susceptibility to allergic diseases [56,57]. A technique of using laminar flow technology to control the temperature around the atopic dermatitis patient can provide some relief to the patient's symptoms [58]. Desiccating stress can alter the immune cell population in the conjunctiva, increasing the expression of proinflammatory genes and encouraging the development of monocytes into antigen-presenting cells [59,60]. At high temperatures, the number of neutrophils has been reported to be elevated, and the mediators released by neutrophils can lead to cell and tissue damage [61-63]. Further studies are needed to verify temperature-health and allergy correlation biological mechanisms.

Several strengths in this study should be highlighted. In our study, it was first shown that humidex was associated with outpatient visits for allergic conjunctivitis in children and adolescents. In addition, we not only obtained the RR and 95% CI of the humidex on allergic conjunctivitis but also investigated the attributional role of the humidex. This study provides an essential reference for a more comprehensive and quantitative study of allergic conjunctivitis and humidity and temperature indices in children. In addition, we selected a relatively long lag period (two weeks). Therefore, we have a longer-term assessment of the exposure relationship between the humidex and allergic conjunctivitis. Moreover, we found a cumulative lag effect of humidex on allergic conjunctivitis. Our study can guide interventions for children with allergic conjunctivitis with different humidex. For example, they should reduce outdoor activities, keep warm, moisturise, etc. The results of the subgroup analysis also suggest that we should pay more attention to the subgroup with a high risk of humidex.

This study also has several limitations. This study is only an urban area, a regional study, and only local meteorological data with patient data in Shanghai were obtained. Further studies on local meteorological conditions are still needed to generalise our findings. Similar to other studies, we could not obtain individual patient exposure data but used average data from fixed meteorological and air pollutant monitoring stations for our analysis. However, because people spend varied amounts of time outside and use different amounts of air cooling, heating, and humidifiers, exposures vary among those with various vocations and from various socioeconomic backgrounds. In addition, there is a lack of comprehensiveness in terms of the scope/coverage of patients and the information collected. We only obtained basic information about the patients, and more detailed personal information about the patients, such as outdoor activity time and lifestyle habits, may impact the correlation study results. A biological basis for the lag days of allergic conjunctivitis visits after exposure to humidity is lacking and may require subsequent basic studies for validation. There are a number of methods to determine the appropriate df and lag days, but subsequently, our research focuses on the possibility of using a function rather than a fixed value and thus performing a time series analysis.

## CONCLUSIONS

In summary, the results of our investigation indicate that exposure to humidex raises the relative risk of children and adolescents with allergic conjunctivitis visits and indicates that humidex exposure brings a specific disease burden. In addition, we found that humidex exposure was more pronounced in boys, those 7-18 years old, and in patients with allergic conjunctivitis during the warm season, a group that requires more attention and interventions. Our study raises awareness of the potential impact of humidex on allergic conditions and related allergic conjunctivitis.

## Additional material


Online Supplementary Document
