# Supplementary material for: Nonlinear effects of humidex on risk of outpatient visit for allergic conjunctivitis among children and adolescents in Shanghai, China: A time series analysis
Source: J Glob Health. 2023 Nov 3;13:04132. doi: 10.7189/jogh.13.04132 (PMC10623378; doi:10.7189/jogh.13.04132)
Supplement: Online Supplementary Document [file jogh-13-04132-s001.pdf]

Online Supplementary Document

Title: Non-linear effects of humidex on risk of hospitalization for allergic conjunctivitis among children and adolescents in Shanghai, China: A time series analysis

Han Zhao[1, 2, 3, 4]* (http://orcid.org/ 0000-0002-5803-505X), Yun Yang[3, 4], Changming Feng[3, 4], Wushuang Wang[3, 4], Chenhao Yang[5], Yue Yin[3, 4], Lan Gong[3, 4]†, Tong Lin[3, 4]† (http://orcid.org/ 0000-0001-5390-2487)

[1] Department of Ophthalmology, Second Xiangya Hospital of Central South University, Changsha, Hunan, 410011, China.

[2] Hunan Clinical Research Center of Ophthalmic Disease, Changsha, Hunan, 410011, China.

[3] Department of Ophthalmology, Eye, Ear, Nose, and Throat Hospital of Fudan University, Shanghai, 200000, China.

[4] Laboratory of Myopia, NHC Key Laboratory of Myopia (Fudan University), Chinese Academy of Medical Sciences, Shanghai, 200000, China.

[5] Department of Ophthalmology, Children's Hospital of Fudan University, Shanghai, 201102, China.

*Joint first authorship.

†Joint senior authorship.

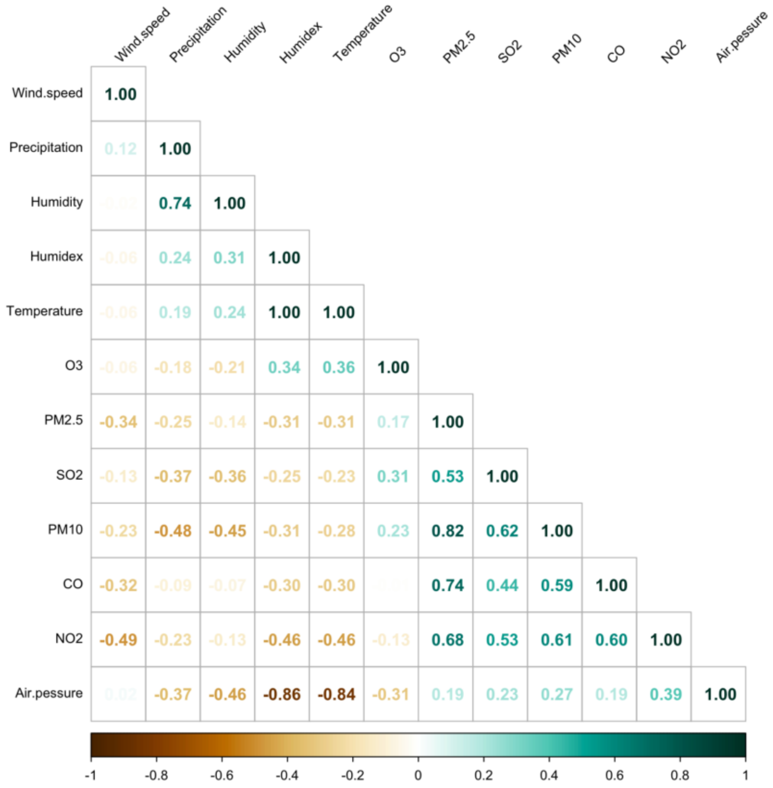

Figure S1. The heatmap shows Spearman's correlation coefficients humidex climate variables, and air pollutants in Shanghai, China, from 1 January 2017 to 31 December 2022.

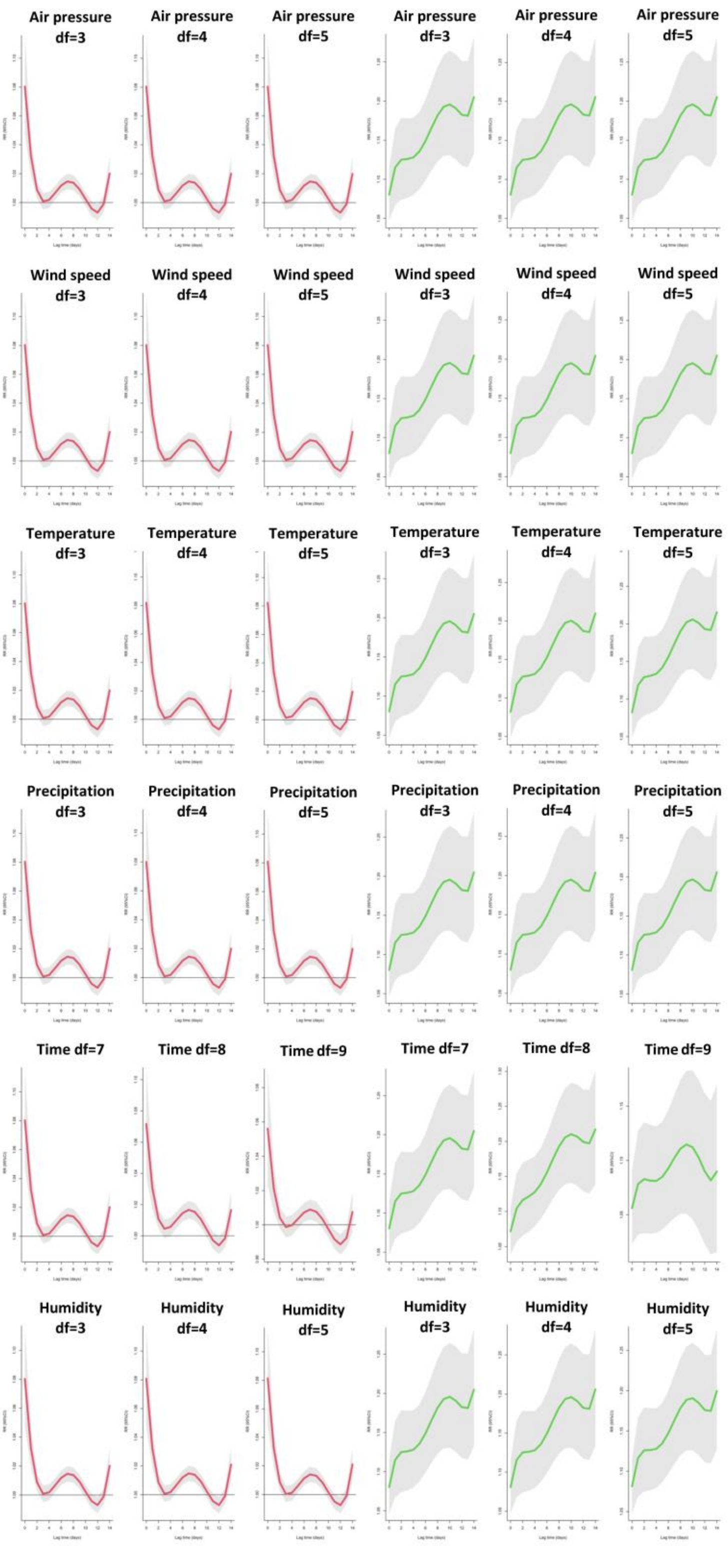

Figure S2. The sensitivity analysis between the various value of df (7-9) for date time and df (3-5) for air pressure, wind speed, mean temperature, precipitation, and humidity on the effects of humidex on child and adolescent allergic conjunctivitis outpatient visits at the single-day lag pattern and cumulative-lag effect pattern.

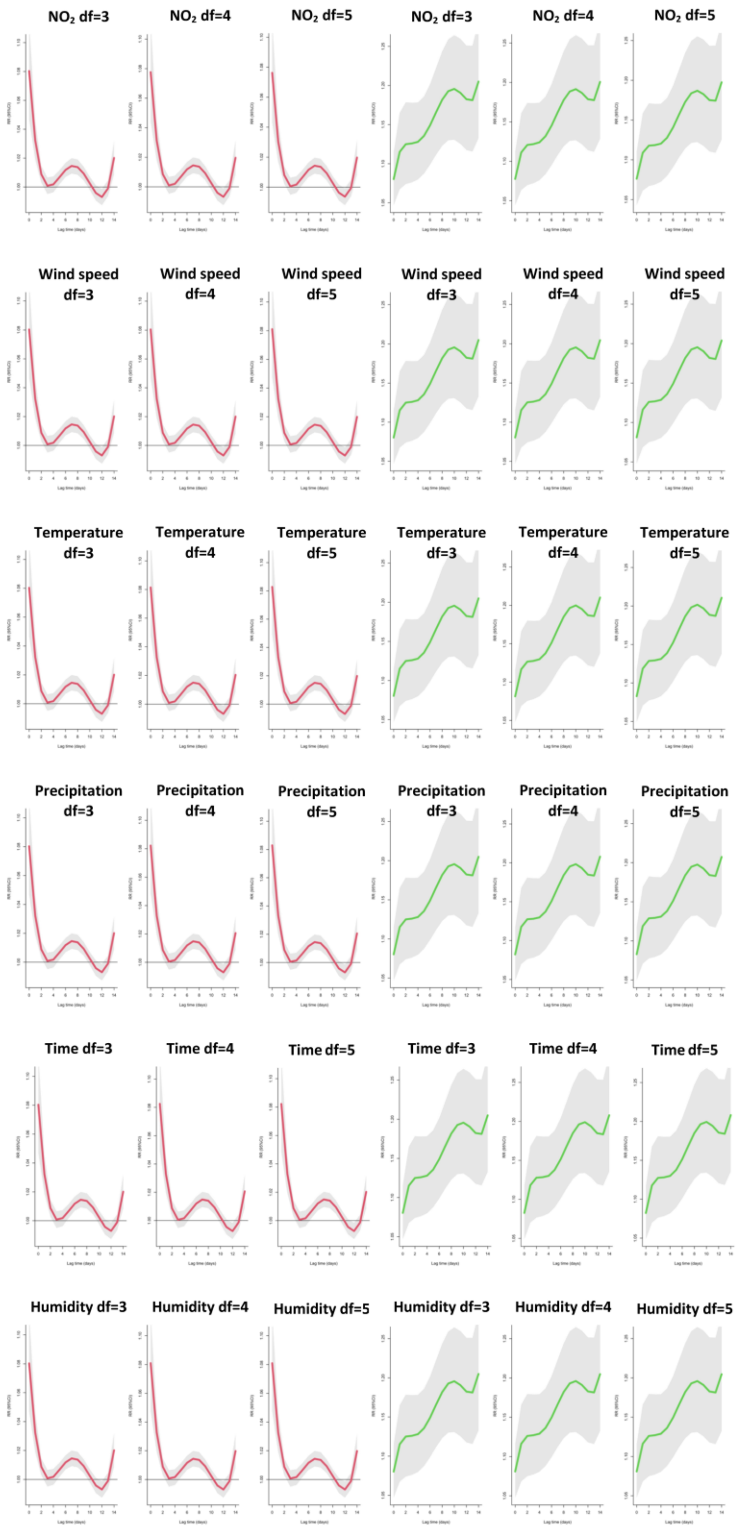

Figure S3. The sensitivity analysis between the various value of df (3-5) for $PM_{2.5}$, PM_{10}, O_3, NO_2, CO, and SO_2 on effects of humidex on the child and adolescent allergic conjunctivitis outpatient visits at the single-day lag pattern and cumulative-lag effect pattern.

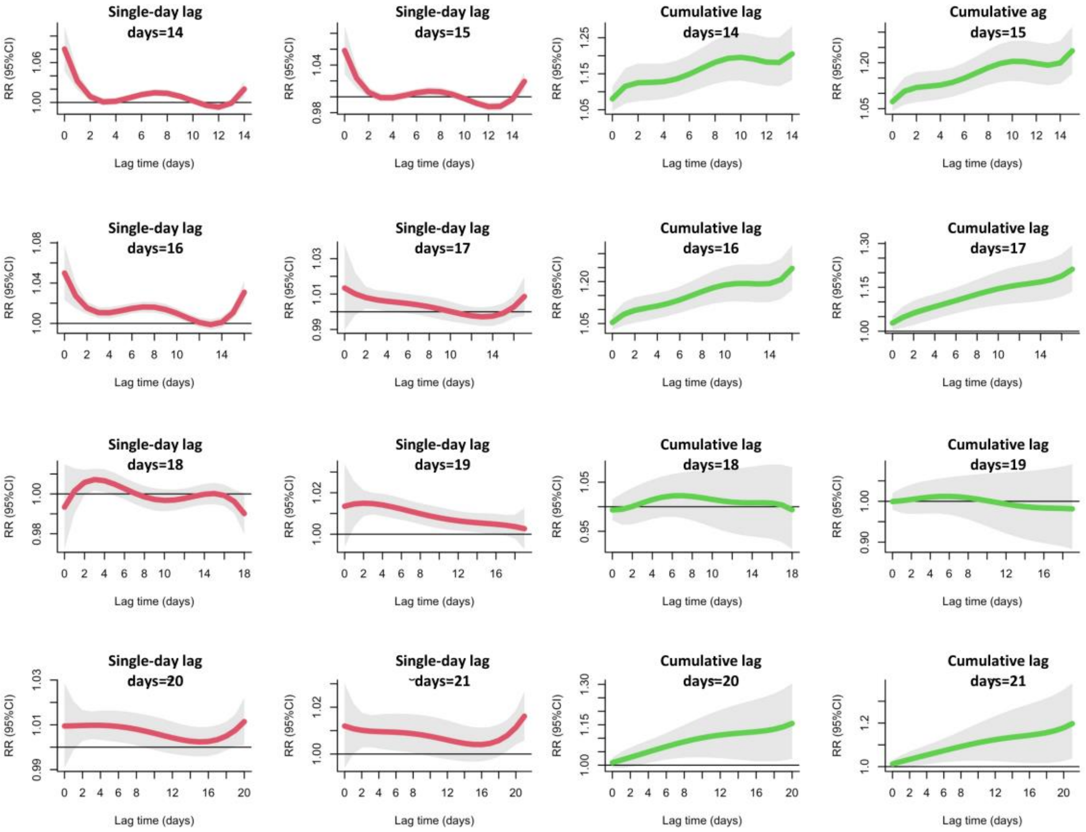

Figure S4. The sensitivity analysis between the changing maximum lag days (14-21) on effects of humidex on the child and adolescent allergic conjunctivitis outpatient visits at the single-day lag pattern and cumulative-lag effect pattern.

Table S1. Summary statistics of humidex categories in Shanghai, 2017–2022.

Groups	Number (Days)	Mean days per year (2017-2022)	Min days/year (Max days/year)
Less than 30°C	1389	277.8	239 days in 2019 (215 days in 2021)
30°C-39°C	428	85.6	91 days in 2021 (54 days in 2022)
40°C-45°C	213	42.6	47 days in 2021 (26 days in 2022)
46°C-54°C	160	32	49days in 2022 (12days in 2021)
Above 54°C	1	0.2	1days in 2017

Table S2. Changes in the relative risk of allergic conjunctivitis for humidex based on the single-day and cumulative lag effects model in total in Shanghai, 2017–2022.

Lag days	Single-day lag RR (95%CI)	Lag days	Cumulative-day lag RR (95%CI)
0	**1.08 (1.05-1.11)**	0-0	**1.08 (1.05-1.11)**
1	**1.03 (1.02-1.05)**	0-1	**1.12 (1.07-1.17)**
2	**1.01 (1.00-1.01)**	0-2	**1.12 (1.07-1.18)**
3	1.00 (0.99-1.01)	0-3	**1.13 (1.08-1.18)**
4	1.00 (0.99-1.01)	0-4	**1.13 (1.08-1.18)**
5	**1.01 (1.00-1.01)**	0-5	**1.14 (1.09-1.19)**
6	**1.01 (1.01-1.02)**	0-6	**1.15 (1.10-1.20)**
7	**1.01 (1.01-1.02)**	0-7	**1.17 (1.11-1.22)**
8	**1.01 (1.01-1.02)**	0-8	**1.18 (1.12-1.24)**
9	**1.01 (1.00-1.01)**	0-9	**1.19 (1.13-1.26)**
10	1.00 (0.99-1.01)	0-10	**1.20 (1.13-1.26)**
11	0.99 (0.99-1.00)	0-11	**1.19 (1.13-1.26)**
12	0.99 (0.98-1.00)	0-12	**1.18 (1.12-1.25)**
13	0.99 (0.99-1.00)	0-13	**1.18 (1.12-1.25)**
14	**1.02 (1.01-1.03)**	0-14	**1.21 (1.13-1.28)**

Table S3. Changes in the relative risk of allergic conjunctivitis for humidex based on the single-day and cumulative lag effects model

in different gender in Shanghai, 2017–2022.

Groups	Lag days	Single-day lag RR (95%CI)	Lag days	Cumulative-day lag RR (95%CI)
Girl	0	**1.07 (1.01-1.12)**	0-0	**1.07 (1.01-1.12)**
	1	**1.02 (1.00-1.05)**	0-1	**1.09 (1.01-1.18)**
	2	1.01 (0.99-1.02)	0-2	**1.10 (1.01-1.19)**
	3	1.00 (0.99-1.01)	0-3	**1.10 (1.02-1.19)**
	4	1.00 (0.99-1.01)	0-4	**1.10 (1.02-1.18)**
	5	1.01 (0.99-1.01)	0-5	**1.11 (1.03-1.19)**
	6	**1.01 (1.00-1.02)**	0-6	**1.12 (1.04-1.21)**
	7	**1.01 (1.00-1.02)**	0-7	**1.13 (1.05-1.23)**
	8	**1.01 (1.00-1.02)**	0-8	**1.15 (1.05-1.25)**
	9	1.01 (0.99-1.02)	0-9	**1.16 (1.05-1.27)**
	10	1.00 (0.99-1.01)	0-10	**1.16 (1.05-1.27)**
	11	0.99 (0.99-1.00)	0-11	**1.15 (1.04-1.27)**
	12	0.99 (0.98-1.00)	0-12	**1.14 (1.03-1.25)**
	13	1.00 (0.99-1.01)	0-13	**1.14 (1.03-1.25)**
	14	1.02 (0.99-1.04)	0-14	**1.16 (1.04-1.28)**
Boy	0	**1.09 (1.05-1.13)**	0-0	**1.09 (1.05-1.13)**
	1	**1.04 (1.02-1.05)**	0-1	**1.13 (1.07-1.19)**
	2	**1.01 (1.00-1.02)**	0-2	**1.14 (1.08-1.21)**
	3	1.00 (0.99-1.01)	0-3	**1.14 (1.08-1.21)**
	4	1.00 (0.99-1.01)	0-4	**1.14 (1.08-1.21)**
	5	**1.01 (1.00-1.01)**	0-5	**1.15 (1.09-1.22)**
	6	**1.01 (1.01-1.02)**	0-6	**1.17 (1.10-1.23)**
	7	**1.02 (1.01-1.02)**	0-7	**1.18 (1.12-1.26)**
	8	**1.01 (1.01-1.02)**	0-8	**1.20 (1.13-1.28)**
	9	**1.01 (1.00-1.02)**	0-9	**1.21 (1.13-1.30)**
	10	1.00 (0.99-1.01)	0-10	**1.22 (1.14-1.30)**
	11	1.00 (0.99-1.00)	0-11	**1.21 (1.13-1.30)**
	12	0.99 (0.99-1.00)	0-12	**1.21 (1.12-1.29)**
	13	1.00 (0.99-1.01)	0-13	**1.21 (1.12-1.29)**
	14	**1.02 (1.01-1.04)**	0-14	**1.23 (1.14-1.33)**

Table S4. Changes in the relative risk of allergic conjunctivitis for humidex based on the single-day and cumulative lag effects model in different age in Shanghai, 2017–2022.

Groups	Lag days	Single-day lag RR (95%CI)	Lag days	Cumulative-day lag RR (95%CI)
0-1 years	0	1.03 (0.89-1.19)	0-0	1.03 (0.89-1.19)

	1	1.00 (0.94-1.06)	0-1	1.03 (0.84-1.26)
	2	1.00 (0.97-1.02)	0-2	1.02 (0.82-1.27)
	3	1.00 (0.98-1.03)	0-3	1.03 (0.83-1.27)
	4	1.02 (0.99-1.05)	0-4	1.05 (0.85-1.28)
	5	**1.03 (1.01-1.06)**	0-5	1.08 (0.88-1.33)
	6	**1.04 (1.02-1.07)**	0-6	1.13 (0.91-1.39)
	7	**1.04 (1.02-1.07)**	0-7	1.18 (0.94-1.47)
	8	**1.04 (1.01-1.06)**	0-8	1.22 (0.96-1.55)
	9	1.02 (1.00-1.04)	0-9	1.24 (0.96-1.61)
	10	1.00 (0.98-1.03)	0-10	1.25 (0.96-1.63)
	11	0.99 (0.96-1.01)	0-11	1.23 (0.94-1.61)
	12	0.98 (0.96-1.01)	0-12	1.21 (0.93-1.58)
	13	0.99 (0.97-1.02)	0-13	1.21 (0.92-1.58)
	14	1.04 (0.98-1.09)	0-14	1.25 (0.93-1.68)
2-6 years	0	1.03 (0.99-1.08)	0-0	1.03 (0.99-1.08)
	1	1.01 (1.00-1.03)	0-1	1.05 (0.99-1.11)
	2	1.00 (1.00-1.01)	0-2	1.05 (0.99-1.12)
	3	1.00 (1.00-1.01)	0-3	1.06 (1.00-1.12)
	4	1.01 (1.00-1.01)	0-4	**1.06 (1.00-1.13)**
	5	**1.01 (1.00-1.02)**	0-5	**1.07 (1.01-1.14)**
	6	**1.01 (1.00-1.02)**	0-6	**1.09 (1.02-1.15)**
	7	**1.01 (1.00-1.02)**	0-7	**1.10 (1.03-1.17)**
	8	**1.01 (1.00-1.02)**	0-8	**1.11 (1.03-1.19)**
	9	1.00 (1.00-1.01)	0-9	**1.11 (1.03-1.20)**
	10	1.00 (0.99-1.00)	0-10	**1.11 (1.03-1.20)**
	11	0.99 (0.99-1.00)	0-11	**1.10 (1.02-1.19)**
	12	0.99 (0.99-1.00)	0-12	**1.10 (1.02-1.18)**
	13	1.00 (1.00-1.01)	0-13	**1.10 (1.02-1.19)**
	14	**1.02 (1.01-1.04)**	0-14	**1.13 (1.04-1.23)**
7-18 years	0	**1.16 (1.10-1.22)**	0-0	**1.16 (1.10-1.22)**
	1	**1.06 (1.04-1.09)**	0-1	**1.23 (1.15-1.32)**
	2	**1.02 (1.01-1.03)**	0-2	**1.25 (1.16-1.35)**
	3	1.00 (0.99-1.01)	0-3	**1.24 (1.16-1.34)**
	4	0.99 (0.99-1.00)	0-4	**1.24 (1.15-1.33)**
	5	1.00 (0.99-1.01)	0-5	**1.24 (1.16-1.33)**
	6	**1.01 (1.00-1.02)**	0-6	**1.25 (1.16-1.34)**
	7	**1.02 (1.01-1.02)**	0-7	**1.27 (1.18-1.37)**
	8	**1.02 (1.01-1.03)**	0-8	**1.29 (1.19-1.40)**
	9	**1.02 (1.01-1.02)**	0-9	**1.31 (1.21-1.43)**
	10	**1.01 (1.00-1.01)**	0-10	**1.32 (1.21-1.45)**
	11	1.00 (0.99-1.01)	0-11	**1.32 (1.21-1.44)**
	12	0.99 (0.98-1.00)	0-12	**1.31 (1.20-1.43)**
	13	0.99 (0.98-1.00)	0-13	**1.30 (1.19-1.42)**
	14	1.01 (0.99-1.03)	0-14	**1.32 (1.19-1.45)**

Table S5. Changes in the relative risk of allergic conjunctivitis for humidex based on the single-day and cumulative lag effects model in warm and cold season in Shanghai, 2017–2022.

Groups	Lag days	Single-day lag RR (95%CI)	Lag days	Cumulative-day lag RR (95%CI)
Warm	0	**1.12 (1.07-1.16)**	0-0	**1.12 (1.07-1.16)**
	1	**1.05 (1.03-1.07)**	0-1	**1.17 (1.10-1.25)**
	2	**1.02 (1.01-1.03)**	0-2	**1.19 (1.12-1.27)**
	3	1.00 (0.99-1.01)	0-3	**1.19 (1.12-1.28)**
	4	1.00 (0.99-1.01)	0-4	**1.19 (1.11-1.27)**
	5	1.00 (0.99-1.01)	0-5	**1.19 (1.10-1.27)**
	6	1.00 (0.99-1.01)	0-6	**1.19 (1.10-1.28)**
	7	1.01 (0.99-1.02)	0-7	**1.20 (1.10-1.31)**
	8	**1.01 (1.00-1.02)**	0-8	**1.21 (1.11-1.33)**
	9	**1.01 (1.00-1.02)**	0-9	**1.23 (1.11-1.36)**
	10	**1.01 (1.00-1.02)**	0-10	**1.24 (1.12-1.38)**
	11	1.00 (0.99-1.01)	0-11	**1.25 (1.12-1.39)**
	12	0.99 (0.98-1.00)	0-12	**1.24 (1.11-1.39)**
	13	0.99 (0.98-1.00)	0-13	**1.22 (1.08-1.38)**
	14	0.98 (0.96-1.00)	0-14	**1.20 (1.05-1.36)**
Cold	0	0.89 (0.85-0.94)	0-0	0.89 (0.85-0.94)
	1	0.99 (0.97-1.01)	0-1	0.88 (0.82-0.94)
	2	**1.04 (1.03-1.05)**	0-2	0.91 (0.85-0.99)
	3	**1.06 (1.04-1.07)**	0-3	0.96 (0.89-1.04)
	4	**1.05 (1.04-1.06)**	0-4	1.01 (0.94-1.09)
	5	**1.03 (1.02-1.04)**	0-5	1.04 (0.96-1.12)
	6	1.00 (0.99-1.02)	0-6	1.04 (0.96-1.13)
	7	0.98 (0.97-1.00)	0-7	1.03 (0.93-1.13)
	8	0.97 (0.96-0.98)	0-8	1.00 (0.90-1.10)
	9	0.97 (0.96-0.98)	0-9	0.97 (0.86-1.08)
	10	0.97 (0.96-0.98)	0-10	0.94 (0.83-1.06)
	11	0.98 (0.97-0.99)	0-11	0.92 (0.82-1.04)
	12	0.99 (0.98-1.01)	0-12	0.92 (0.81-1.04)
	13	1.00 (0.99-1.01)	0-13	0.92 (0.80-1.05)
	14	0.99 (0.97-1.01)	0-14	0.90 (0.78-1.05)
